# Mindful self-compassion to reduce stigma among individuals diagnosed with lung cancer (MSC-LC): a pilot study protocol for a parallel-group, randomized controlled trial

**DOI:** 10.1186/s40814-026-01808-8

**Published:** 2026-03-20

**Authors:** Timothy J. Williamson, Whitney M. Brymwitt, Jamie S. Ostroff, Lisa Carter-Bawa, Christopher K. Germer, Steven D. Hickman, Jun J. Mao, Heidi A. Hamann, Kristen E. Riley, Jamie L. Studts, McKenzie T. Reese

**Affiliations:** 1 Department of Psychological Science, Loyola Marymount University, 1 LMU Drive, Suite 4700, Los Angeles, CA 90045 USA; 2 Department of Psychiatry and Behavioral Sciences, Memorial Sloan Kettering Cancer Center, New York, NY USA; 3 Cancer Prevention Precision Control Institute, Center for Discovery & Innovation at Hackensack Meridian Health, Nutley, NJ USA; 4 Georgetown Lombardi Comprehensive Cancer Center, Washington, D.C. USA; 5 Department of Psychiatry, Harvard Medical School, Cambridge, MA USA; 6 Center for Mindfulness, University of California, San Diego, CA USA; 7 Department of Medicine, Integrative Medicine Service, Memorial Sloan Kettering Cancer Center, New York, NY USA; 8 Department of Psychology, University of Arizona, Tucson, AZ USA; 9 Graduate School of Applied and Professional Psychology, Rutgers, The State University of New Jersey, Newark, NJ USA; 10 Department of Medicine, University of Colorado School of Medicine, Aurora, CO USA

**Keywords:** Lung cancer, Stigma, Self-compassion, Mindfulness, Pilot trial, Mixed-methods

## Abstract

**Background:**

Lung cancer stigma (i.e., perceived and internalized negative appraisal and devaluation associated with lung cancer) is a clinically significant psychosocial concern among individuals diagnosed with lung cancer. Stigma has been linked to increased psychological distress, higher depressive symptoms, anxiety, lower quality of life, and reduced engagement in care. However, few patient-centered interventions address lung cancer stigma. Mindful Self-Compassion (MSC) is a promising approach that promotes emotional resilience and reduces psychological distress. However, MSC has not been tailored for the needs of people with lung cancer. To address this gap, we developed Mindful Self-Compassion for Lung Cancer (MSC-LC)—a disease-specific adaptation designed to reduce stigma by cultivating self-compassion. This study aims to assess the feasibility, acceptability, and preliminary efficacy of MSC-LC among individuals with lung cancer who report elevated stigma.

**Methods:**

Theoretically grounded in the Conceptual Model of Lung Cancer Stigma, the Health Stigma and Discrimination Framework, and the CDC Map of Adaptation, this pilot trial uses a parallel-group randomized controlled design (1:1 allocation) comparing MSC-LC to an enhanced standard-of-care waitlist control group (CTL). We aim to enroll 60 adults diagnosed with lung cancer (*n*=30 per condition) who exceed a validated cutoff for elevated stigma on the Lung Cancer Stigma Inventory. MSC-LC includes weekly 90-min virtual group sessions co-facilitated by trained interventionists and home practice assignments between sessions. Feasibility and treatment fidelity (primary outcomes) will be assessed using quantitative benchmarks (e.g., recruitment, retention, session attendance, interventionist adherence). Acceptability will be evaluated through qualitative interviews. Secondary outcomes including stigma, self-compassion, depressive symptoms, anxiety, and spiritual well-being will be collected at baseline, mid-intervention, post-intervention, and longer-term follow-up. Quantitative and qualitative data will be integrated at the interpretation phase to examine signals of change and inform refinement and implementation.

**Discussion:**

This study will address the gap of patient-centered interventions that target the reduction of lung cancer stigma. Findings will inform the rigorous evaluation of the efficacy of MSC-LC to reduce stigma among individuals with lung cancer.

**Trial registration:**

The protocol for this study has been registered on ClinicalTrials.gov (ID: NCT06191939). First posted December 20, 2023, last update posted July 17, 2025. https://clinicaltrials.gov/study/NCT06191939.

## Background

Adults diagnosed with lung cancer frequently report experiences of stigma (i.e., perceived and internalized negative appraisal and devaluation associated with lung cancer), a critical psychosocial issue that contributes to greater distress, higher depressive symptoms, higher anxiety, worse quality of life, greater sleep disruption, and more bothersome physical symptoms [1–7]. Importantly, lung cancer stigma often carries a moral dimension, rooted in perceptions of personal responsibility or blame (e.g., attribution theory) [8], which can intensify distress and further marginalize people diagnosed with lung cancer [9]. Reduction of stigma for this vulnerable and growing patient population is an urgent need [10, 11]. Despite these associations, there is a gap in the field regarding empirically supported, patient-focused interventions that target the reduction of lung cancer stigma [12, 13].

To address this need, we developed Mindful Self-Compassion for Lung Cancer (MSC-LC)—a disease-tailored, mindfulness-based psychosocial intervention that aims to reduce lung cancer stigma by cultivating self-compassion skills [14]. MSC-LC was developed through a rigorous, multi-phase adaptation process to enhance relevance, accessibility, and safety for individuals with lung cancer [14]. The intervention builds upon the empirically supported Mindful Self-Compassion (MSC) program, an 8-week, group-based program that has demonstrated efficacy in increasing self-compassion and reducing shame, self-criticism, anxiety, and depression in non-cancer populations [15–20].

Self-compassion is defined as directed kindness towards oneself in times of suffering [21], and it is increasingly recognized as a protective psychosocial factor in the context of chronic illness [18, 19]. Among adults with lung cancer, higher levels of self-compassion have been shown to buffer the association between stigma and depression [22], suggesting it may serve as an effective psychological target for intervention. However, given several anticipated challenges associated with standard delivery of MSC to lung cancer patients (e.g., breathing challenges that arise during breath-focused meditations, fatigue that interferes with attending 3-hour sessions), the investigators created MSC-LC to better meet the needs of people living with lung cancer [14, 23–25]. Informed by perspectives from individuals with lung cancer, psycho-oncology clinicians, expert MSC teachers, and stigma researchers, major adaptations include a shorter session length, modified breath- and movement-focused practices, simplified home exercises, and explicit inclusion of content that acknowledges, explores, and responds to the experience of lung cancer stigma [14].

Despite the plausibility of self-compassion as an intervention target and the rigorous adaptation process of developing MSC-LC, no randomized controlled trials (RCTs) to date have tested the feasibility of a group-based psychosocial intervention designed to reduce lung cancer stigma. As such, it is not yet known whether MSC-LC is acceptable to individuals diagnosed with lung cancer, feasible to deliver, and associated with meaningful reductions in stigma and related distress.

### Aims of the current study

The primary aim of this pilot RCT is to evaluate the intervention feasibility and treatment fidelity and acceptability of the MSC-LC program in adults with lung cancer who report clinically elevated stigma. The target for trial feasibility is 50% of eligible participants agreeing to enroll in the study and be randomized (recruitment), 30% attrition or lower (retention), and 60% of participants in the MSC-LC condition attending six or more session (attendance). The target for treatment fidelity is >80% of intervention components deliver per session (interventionist adherence to protocol) and average intervention delivery competency ratings greater than or equal to 4 out of 6 (indicating “competent” delivery) using a standardized assessment. Acceptability will be evaluated through qualitative feedback on appropriateness and satisfaction of the MSC-LC intervention.

This pilot trial will also explore whether MSC-LC is associated with improvements in lung cancer stigma, self-compassion, depressive symptoms, anxiety, and spiritual well-being (secondary outcomes), and examine theory-driven mechanisms of change. In addition, we will conduct post-intervention qualitative interviews with participants to identify salient experiences, mechanisms of change, and areas for refinement not captured in quantitative outcomes.

## Methods

### Study design

Theoretically grounded in the Conceptual Model of Lung Cancer Stigma, the Health Stigma and Discrimination Framework, and the CDC Map of Adaptation [26–28], the current study is a pilot, mixed-methods, parallel group RCT where participants are randomized with a 1:1 ratio to either the MSC-LC intervention (a 10-week, virtually-delivered, group-based psychosocial intervention focused on the development of mindfulness and self-compassion skills) or to an enhanced standard-of-care waitlist control group (CTL). The reporting of this protocol is aligned with the Standard Protocol Items Recommendations for Interventional Trials (SPIRIT) guidelines [29]. All procedures were approved by the Institutional Review Board (IRB) at Loyola Marymount University. Any protocol amendments will be submitted and approved by the IRB prior to making any changes to the study procedures.

### Participant eligibility

Inclusion criteria for participant eligibility include (1) being at least 18 years of age; (2) having a confirmed diagnosis of lung cancer, as per self-report and verified by treating clinician or medical record; (3) a score of 38 or higher (above the validated cutoff for clinically significant stigma burden) on the Lung Cancer Stigma Inventory (LCSI) [1, 30]; and (4) ability to read and respond to questions in English. Exclusion criteria include (1) inability to understand study procedures or the informed consent process, or observable interpersonal behaviors documented during screening (e.g., hostility or antagonism, violations of interpersonal boundaries) that would interfere with safe and constructive participation in a group-based intervention, as determined by the study clinician (Principal Investigator) in consultation with the screening research team member; (2) completed a course of Mindful Self-Compassion or an equivalent meditation training in the prior 12 months; and (3) use of antidepressant, anxiolytic, antipsychotic, or mood stabilizing medication(s) for which the dose has been initiated or changed within the 8 weeks prior to study entry. To accommodate the complex and individualized needs of people with lung cancer, participants will be permitted to continue their usual medical and psychological care during their study participation. The only restrictions are concurrent or recent (within the past 12 months) enrollment in another course focused on mindful self-compassion or similar mindfulness-based training (e.g., Mindfulness-Based Stress Reduction).

### Participant recruitment, screening, informed consent, and randomization

Our national recruitment approach will engage multiple strategies, including sharing study information with clinics, lung cancer survivorship groups, and non-profit organizations across the USA; direct messaging (via email and text) to existing registries of people who are interested in participating in clinical trials (e.g., ClinicalConnection); and direct-to-participant recruitment via social media and other web-based platforms [31–33]. We will contract with a third-party vendor (BuildClinical, LLC) to assist with direct-to-participant recruitment [34, 35]. BuildClinical deploys study-specific advertisements to engage participants on various social media and other digital platforms (e.g., Facebook, Google, WebMD). When individuals click the advertisement (e.g., “Join a Remote Study to Improve Lung Cancer Care”), they will be redirected to a study-specific landing page with study procedures, risks, benefits, and compensation. On the landing page, individuals are invited to complete a pre-screening questionnaire online (assessing age, diagnosis of lung cancer, and comfort responding to questions in English).

**Fig. 1:**
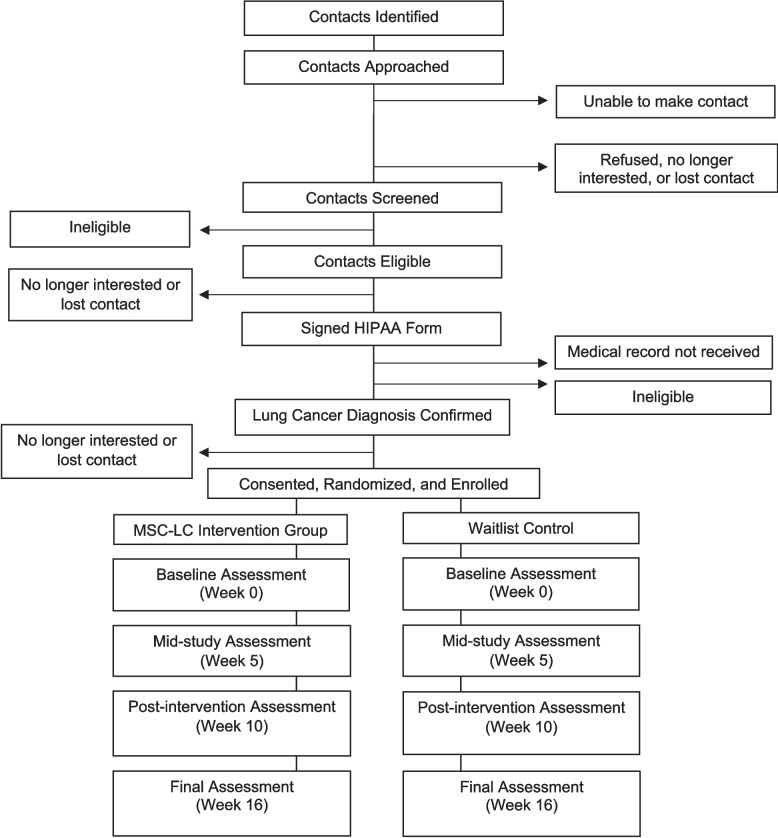
Schedule of enrollment, treatment, and data collection

Interested and potentially eligible participants provide authorization to BuildClinical to provide their name and contact information to the study team. Potential participants recruited via other methods (e.g., outreach from a local non-profit organization) will be directed to a similar web-based form to complete a pre-screening questionnaire and provide their contact information. Then, the study team will contact potential participants via phone to complete a screening survey (verifying pre-screening responses and assessing study inclusion and exclusion criteria). All participants will be assigned a study participant ID at the onset of screening (prior to randomization), which will be used to facilitate questionnaire completion and de-identified data management. An overview of participant flow from initial contact through study completion is presented in Fig. 1.

Individuals confirmed as interested and eligible will then complete and digitally sign a HIPAA authorization for the release of protected health information, which authorizes the individual’s oncology care clinician to confirm the individual’s lung cancer diagnosis to the study team. The study team will confirm the participant’s diagnosis by reviewing a medical record note, which can be shared by the oncology care clinician via fax, mail, secure digital message or from by participant via self-uploading a file to a secure web-based form. All medical record notes will be destroyed promptly after confirming the participant’s diagnosis.

After confirming the participant’s diagnosis, the study team will contact the participant via secure teleconferencing software (e.g., Zoom) to maintain participant protections. To verify the participant’s identity, participants will be asked to present photo identification to the study team member for visual verification (no record of the photo identification will be retained by the study team). The study team member will verbally explain the study procedures, risks, benefits, compensation, and all information in the informed consent form. All participants will provide signed informed consent (digitally). All participants are provided with information for 24-h crisis support services (e.g., 988) at study onboarding and reminded of these resources as needed. Throughout the study, participants who experience psychological distress may contact the on-call study clinician (Principal Investigator) during working hours.

After providing signed informed consent, participants will be randomized to either the MSC-LC or CTL condition. Prior to randomization, a randomized allocation sequence will be generated using the NCI Clinical Trial Randomization Tool [36]. Parameters for generating the allocation sequence include an asymptotic maximal randomization method, a maximum tolerated imbalance of 2, and an allocation ratio of 1:1 without stratification. Randomization will not be stratified in this pilot study, informed by formative qualitative findings indicating participant preference for heterogeneous group composition and expert guidance emphasizing the importance of shared emotional experiences across varying clinical contexts (e.g., disease stage, treatment) within the MSC-LC intervention [14]. To prevent selection bias, the research staff is provided with a participant’s allocation only after that participant has provided informed consent and has been accepted into the trial. Because the MSC-LC intervention is delivered in group format, randomization occurs prior to baseline assessment to allow for scheduling alignment among those assigned to the intervention group. Due to the nature of the behavioral intervention, participants and interventionists cannot be blinded to condition assignment.

### Data management and access

Data management and access will be restricted to study personnel approved by the Institutional Review Board. All data will be stored on secure, password-protected servers and encrypted in accordance with institutional data protection policies. Only the Principal Investigator and IRB-approved study team members will have access to the final trial datasets, including identifiable information, which will be stored separately from study data, linked through the participant study ID, and used solely for recruitment, scheduling, and follow-up communications. All individuals with access will be trained in confidentiality protocols and responsible data handling procedures. De-identified datasets will be used for analysis.

A formal Data Monitoring Committee is not planned due to the minimal-risk nature of this single-site, behavioral intervention [37]. Trial conduct and participant safety will be monitored by the Principal Investigator and Project Manager, who will independently and jointly review recruitment, retention, adherence, and any safety concerns on an ongoing basis.

### Sample size justification

Consistent with methodological guidance that pilot trial sample size should be large enough to estimate key feasibility parameters with acceptable precision, minimum sample size was determined based on feasibility objectives and progression criteria rather than testing for intervention efficacy [38].

A priori progression criteria were defined for recruitment, retention, session attendance in the MSC-LC condition, and treatment fidelity. Recruitment will be considered feasible if at least 50% of eligible participants enroll and not feasible if recruitment uptake is 33% or less. Retention will be considered feasible if 75% or more of participants complete follow-up assessments and not feasible if retention is 50% or less. MSC-LC attendance will be considered feasible if at least 60% of participants attend six or more sessions and not feasible if 33% or fewer attend six or more sessions. Treatment fidelity will be considered feasible if at least 80% of intervention components are delivered per session and not feasible if delivery is 50% or less. Using the normal approximation method for a two-arm parallel design with one-sided *α* =.05 and 90% power to evaluate these feasibility thresholds [38], the *minimum* required sample size was *n* = 62 (*n* = 31 per condition).

MSC-LC will be delivered across three planned cohorts, each designed to include approximately 10–15 participants to facilitate meaningful discussion and interpersonal exchange [14, 39]. Accordingly, total recruitment may range from 62 to 90 participants to ensure adequate group size across cohorts. Although the sample size is consistent with accepted conventions for pilot trials, we acknowledge that it limits the precision and generalizability of the quantitative findings. The analyses are intended to inform the design and feasibility of a future fully powered trial.

### Interventionists

Each cohort of the MSC-LC intervention will be delivered by two Certified MSC teachers who have completed the core MSC curriculum, a 12-week Teacher Training, and a 10-week Teacher Training Practicum, have co-taught the MSC curriculum with consultation from a senior MSC teacher, subsequently taught at least three additional MSC courses, and received at least 14 h of individual mentorship from a senior MSC teacher over the course of teaching an additional two MSC courses [40]. The interventionists will also receive a 90-min training on the MSC-LC adaptation, which includes an overview of lung cancer stigma definitions and examples, relevant research on tailoring MSC to this population, as well as guidance on modifying language and offering flexible options for breathwork and movement practices to better meet participants’ needs and lived experiences [14]. Interventionists will also participate in weekly supervision with the PI to review participant concerns and support trauma-sensitive and culturally responsive intervention delivery.

### Intervention description

#### Mindful Self-Compassion for Lung Cancer (MSC-LC)

MSC-LC is a group-based psychosocial intervention that consists of 10 consecutive weekly 90-min sessions, facilitated by two trained interventionists. The overall goal of the intervention is to enhance recognition and responsiveness to difficult thoughts and emotions (particularly those arising from their diagnosis of lung cancer and any associated stigma) and to build self-compassion skills that facilitate patients’ ability to turn inward to those thoughts and feelings with mindfulness, connectedness, and self-kindness. The treatment manual is adapted based on the Mindful Self-Compassion Teacher Guide from the Center for Mindful Self-Compassion [14, 39].

Each session follows an agenda with highly structured scripts covering self-compassion topics, which are carefully organized to build upon one another (Table 1). Specifically, the program begins with introductions and setting group expectations before sequentially building core MSC-LC skills focusing on mindfulness, kindness and compassion for others, kindness and compassion toward oneself, managing difficult emotions, responding to distress that arises from lung cancer stigma, navigating challenging relationships, and a final reflection and integration session. A silent retreat session midway through the intervention program offers an uninterrupted opportunity for intensive practice of core skills. Each session includes didactic or psychoeducational topics (e.g., “what is self-compassion?”), experiential activities, guided meditations, group discussion (both in the full group and within pairs or trios), and home practice assignments to reinforce learning. Sessions are delivered in small groups (typically 8–12 participants), and participants are asked to join virtually from a private space to support a confidential and supportive group environment. Sessions are co-facilitated by two trained interventionists to allow brief individualized support in a private breakout room for participants experiencing heightened distress, with follow-up contact from the study team and reminders of available support resources as needed.

**Table 1:** Overview of sessions in Mindful Self-Compassion for Lung Cancer (MSC-LC)

Session	Psychoeducational/Didactic	Skills/Meditation	Home Practice
Session 1: Introduction to Mindful Self-Compassion	• Introductions • Group expectations • What is Self-Compassion? • How to approach MSC	• Why Am I Here? • How Would I Treat a Friend? • Self-Compassion Break • Soothing and Supportive Touch	• Self-Compassion Break • Soothing and Supportive Touch
Session 2: Practicing Mindfulness	• Practicing Mindfulness • What is Mindfulness? • What are Backdraft and Resistance? • Relationship between Mindfulness and Self-Compassion	• Affectionate Breathing • Soles of the Feet • Mindfulness & Self-Compassion in Daily Life	• Affectionate Breathing and Swaying • Soles of the Feet • Mindfulness in Daily Life • Self-Compassion in Daily Life
Session 3: Practicing Kindness	• Relationship between Kindness and Compassion • Practicing with Phrases	• Affectionate Breathing • Awakening Our Hearts • Loving-Kindness for a Loved One • Finding Loving-Kindness Phrases	• Loving-Kindness for a Loved One • Finding Loving-Kindness Phrases
Session 4: Discovering Your Compassionate Voice	• Discovering your Compassionate Voice • MSC Stages of Progress • Self-Criticism and Safety	• Loving-Kindness for Ourselves • How is MSC-LC Going for Me? • Motivating Ourselves with Compassion	• Loving-Kindness for Ourselves • Compassionate Letter to Myself
Session 5: Living Deeply	• Living Deeply • Core Values • Finding Meaning Amidst Suffering	• Giving and Receiving Compassion • Discovering Our Core Values • Moments of Meaning	• Compassionate Listening • Living with a Vow • Giving and Receiving Compassion
Session R: Retreat	• How to approach the Retreat • Posture Instruction	• Affectionate Breathing • Loving-Kindness for Ourselves • Compassionate Movement • Giving and Receiving Compassion • Coming out of Silence	• Sense and Savor Walk • Compassionate Movement • Walking in Loving-Kindness • Savoring Food • Compassionate Body Scan
Session 6: Meeting Difficult Emotions	• Meeting Difficult Emotions • Approaches to Difficult Emotions • Introduction to Shame	• Loving-Kindness for Ourselves • Being with Difficult Emotions • Self-Compassion Break for Shame	• Being with Difficult Emotions • Self-Compassion Break for Shame • Working with Shame
Session S: Stigma and Self-Compassion	• Stigma, Mindfulness, and Self-Compassion • Fierce Self-Compassion for Stigma	• Giving and Receiving Compassion • Mindfulness of Stigma in Daily Life • The Wish to be Loved • Fierce Self-Compassion Break for Stigma	• Fierce Self-Compassion Break for Stigma
Session 7: Exploring Challenging Relationships	• Exploring Challenging Relationships • Pain of Disconnection • Pain of Connection	• Compassionate Friend • Anger and Unmet Needs • Compassion with Equanimity	• Compassionate Friend • Self-Compassion Break in Relationships • Compassion with Equanimity
Session 8: Embracing Your Life	• Embracing Your Life • Cultivating Happiness • Savoring and Gratitude • Self-Appreciation	• Compassion for Self and Others • Gratitude for Small Things • Appreciating Our Good Qualities • What would I like to Remember?	• Compassion for Self and Others • Gratitude for Small Things • Appreciating Our Good Qualities

Participants will receive guidance in advance regarding virtual platform access and basic troubleshooting, and a technology check will be conducted for each participant prior to the first session. During each session, a member of the research team will be available in real time to assist with connectivity, audio, or video issues and to support participants in rejoining sessions promptly if disruptions occur.

After each session, participants receive educational materials summarizing session content and home practices, which are provided in the formats of a digital handout (PDF) and audio recording (MP3). At the end of the trial, participants will be provided with a restricted-access folder that contains all the home practice materials (audio files, PDF handouts); otherwise, no ancillary or post-trial care is provided.

To promote adherence to the intervention protocol, all MSC-LC sessions will be audio-recorded, with participant consent. Weekly supervision meetings with interventionists will be led by the PI (TJW) to review recordings, provide feedback, and address any deviations from the protocol. Interventionists will utilize structured session agendas and checklists to standardize delivery and ensure all key MSC-LC components are covered. Home practice is encouraged through discussion in sessions, reminder emails, and weekly participant logs.

Participants may withdraw from the intervention at any time by choice or may be asked to discontinue by the study team if continued participation risks negatively influencing group dynamics or the individual’s well-being, per study clinician’s judgment. This will be assessed weekly through review of audio-recorded intervention sessions and discussions between the interventionists and the PI (TJW), who is a licensed clinical psychologist.

#### Enhanced standard of care with waitlist control group (CTL)

At present, there are no established clinical guidelines specifically addressing stigma-related distress in individuals with lung cancer. However, the National Comprehensive Cancer Network (NCCN) recommends referral to mental health services for cancer patients experiencing heightened distress. In alignment with these recommendations, participants randomized to the enhanced standard of care with waitlist control group (CTL) will receive an information sheet developed by the National Cancer Institute, which includes guidance on accessing supportive resources and organizations in their community.

Control group participants will also be placed on a waitlist to receive the MSC-LC intervention, and participants are explicitly told they are on a waitlist. Following completion of all scheduled study assessments, they will be offered the opportunity to receive the MSC-LC intervention. This waitlist design ensures that all participants eventually have access to the intervention, while also enabling comparison of the MSC-LC intervention to an enhanced version of current standard care. This approach will allow for rigorous evaluation of whether the MSC-LC intervention provides benefits beyond existing practices for managing distress in people with lung cancer.

### Participant retention

Several strategies will be used throughout the pilot trial to promote participant retention and minimize attrition. During the consent process, participants will be informed of the importance of completing all sessions and questionnaire assessments. Email and text reminders will also be sent to MSC-LC and CTL participants at the beginning of each assessment window. Additionally, email and text reminders will be sent to MSC-LC participants prior to each group session along with mid-week follow-up reminders about home practice.

Attendance will be monitored throughout the program to support retention and identify potential barriers to participation. MSC-LC participants will be asked to inform the study team in advance if they expect to miss a session, and a study team member will follow up by phone if a participant is unexpectedly absent. If a participant misses a session without notifying the study team in advance, a study team member will initiate follow-up contact via email, text message, and phone call to check in, assess any concerns, and collaboratively problem-solve barriers to attendance (e.g., technology difficulties, reminder preferences, scheduling challenges, or health-related issues). Participants are reminded during these interactions that participation in the intervention and study is voluntary and that they may withdraw at any time. Participants may miss no more than five sessions across the intervention period to continue participating in the MSC-LC group. Importantly, participants who miss more than five sessions will be invited to continue completing study questionnaires.

### Data collection

Following the informed consent procedures described above, participants complete an initial questionnaire (T0) that includes demographic and medical information, including smoking history. The baseline assessment (T1) is sent 1 week prior to the first group session and must be completed before the first session. All subsequent questionnaires are administered at three additional timepoints: mid-intervention (T2; 5 weeks after baseline), post-intervention (T3; 10 weeks after baseline), and longer-term follow-up (T4; 16 weeks after baseline). This schedule of assessments is the same across both the MSC-LC and CTL groups. Weekly home practice surveys are also distributed to MSC-LC participants the day before each group session to capture their engagement with home practice exercises during the prior week. All questionnaires are administered electronically via Qualtrics.

Within 2 months after the conclusion of the intervention, all MSC-LC participants who attended at least 1 session will be invited to participate in an individual, semi-structured qualitative interview with a member of the study team.

Participants may receive up to $150 for completing the four assessment timepoints ($37.50 per completed assessment), with compensation provided as a single e-gift card at the conclusion of the study. Participants who complete a post-intervention qualitative interview will receive an additional $60 e-gift card.

### Study measures

The study includes both early implementation process outcomes and patient-reported outcomes. Implementation measures include intervention feasibility and treatment fidelity. Patient-reported outcomes will be collected via Qualtrics at study enrollment (T0), baseline (T1; when the MSC-LC intervention begins), mid-intervention (T2; 5 weeks after baseline), post-intervention (T3; 10 weeks after baseline), and longer-term follow-up (T4; 16 weeks after baseline). See Table 2 for a complete list of all study measures and their schedule of assessment. Measures were selected based on prior qualitative research identifying these constructs as central to experiences of stigma and coping [14]. Anticipated survey durations (approximately 45 min at baseline and 30 min at follow-up) have been shown to be acceptable in similar populations and were also endorsed as reasonable during formative qualitative work.

**Table 2:** Schedule of participant assessments

Construct	Measure	Number of Items	Study Entry (T0)	Baseline (T1)	Mid-Intervention (T2)	Post-Intervention (T3)	Longer-Term Follow-Up (T4)
Patient-Reported Outcomes							
Lung cancer stigma	Lung Cancer Stigma Inventory	25		x	x	x	x
Self-Compassion	Self-Compassion Scale	26		x	x	x	x
Depressive symptoms	Patient Health Questionnaire-8	8		x	x	x	x
Anxiety	Generalized Anxiety Disorder-7	7		x	x	x	x
Spiritual well-being	Functional Assessment of Chronic Illness Therapy-Spiritual Well-Being, meaning and peace subscales	8		x	x	x	x
Mediators							
Sleep disturbances	Patient-Reported Outcomes Measurement Information System-Sleep Disturbance, short form 8b	8		x	x	x	
Physical symptom burden	Edmonton Symptom Assessment System-revised, physical symptoms subscale	6		x	x	x	
Emotional Responses to Dyspnea	Multidimensional Dyspnea Profile, breathing discomfort and emotional response subscales with serenity adjectives from the PANAS-X	8		x	x	x	
Social Attachment	Social Provisions Scale, attachment subscale	4		x	x	x	
Loneliness	UCLA Loneliness Scale-3	3		x	x	x	
Rumination	Rumination-Reflection Questionnaire, rumination subscale	12		x	x	x	
Mindfulness	Five Facet Mindfulness Questionnaire, observing subscale	3		x	x	x	
Sample Characteristics and Covariates							
Demographic characteristics	Demographic Information	12	x				
Medical Information	Medical Information	6	x				
Tobacco history	The Cancer Patient Tobacco Use Questionnaire (modified version)	7	x				
General health	Patient-Reported Outcomes Measurement Information System, Global Health-Physical	1		x	x	x	x

After providing informed consent, participants complete an initial questionnaire (T0). The baseline assessment (T1) is sent one week prior to the first group session and must be completed before the first session. All subsequent questionnaires are administered at three additional timepoints: mid-intervention (T2; 5 weeks after baseline), post-intervention (T3; 10 weeks after baseline), and longer-term follow-up (T4; 16 weeks after baseline). The schedule of assessments is the same across participants in the intervention and control groups.

#### Sociodemographic and medical characteristics

Sociodemographic data collected via self-report will include age, gender, race, ethnicity, education, employment, insurance status, household income and savings, cancer-related medical debt, and out-of-pocket costs in the past 6 months. Participant zip codes will also be collected to facilitate potential linkage of individual-level data with community-level social drivers of health data. Medical information gathered by self-report will include lung cancer type and stage, time since diagnosis, oncologic treatments, and smoking history assessed with the Cancer Patient Tobacco Use Questionnaire (C-TUQ, modified version) [41]. General health will be assessed using the single-item Patient-Reported Outcomes Measurement Information System (PROMIS) Global Health-Physical scale, in which participants will respond on a 5-point Likert scale to indicate their overall health from 1 (“poor”) to 5 (“excellent”) [42].

#### Intervention feasibility and treatment fidelity (primary outcomes)

Feasibility will be assessed using three indicators, with the number of MSC-LC sessions each participant attends reflecting feasibility of the intervention itself and the number of eligible patients who enroll in the study (recruitment) and the number of questionnaire assessments completed by both MSC-LC and CTL participants (retention) reflecting feasibility of the research protocol for potential scale-up to a larger trial [43]. A priori benchmark indicators to assess feasibility (and treatment fidelity) are listed below in the Data Analysis plan.

Treatment fidelity will be measured via two indicators: checklist adherence and qualitative ratings of interventionist competence. Adherence will be assessed as the proportion of predefined session components delivered by interventionists, based on review of session audio recordings [44]. Two trained study team members will review each recording, and weekly consensus meetings will be held to determine final ratings. This measure of adherence reflects the extent to which key elements of the intervention were implemented as intended [44]. Fidelity via interventionist competence will be evaluated using the MSC Teaching Assessment Criteria (MSC TAC) [45], which is an adapted version of the validated Mindfulness-Based Interventions – Teaching Assessment Criteria tool [46]. The MSC TAC is a structured tool to assess interventionist competence in the delivery of MSC across seven domains (understanding the curriculum, embodying self-compassion, relating compassionately to others, teaching topics, guiding practices and class exercises, facilitating group process, engaging in inquiry) using a 6-point adjectival scale of competence (incompetent, beginner, advanced beginner, competent, proficient, advanced). A rater with expert training and extensive experience in using the MSC TAC to evaluate interventionist competence will review audio recordings to independently rate 3 out of 10 sessions per MSC-LC cohort, with one session randomly selected each from the early (weeks 1–3), middle (weeks 4–6), and late (weeks 7–10) phases of the intervention. The rater will be blinded to whether the audio is from the first, second, or third MSC-LC cohort. Given the extensive familiarity of the MSC curriculum needed to be trained on using the MSC TAC, the rater cannot be fully blinded to intervention timing (early, middle, late). This measure of competence reflects the skill with which the interventionists embodied and delivered the core principles and practices of MSC.

#### Patient-reported outcomes (secondary outcomes)

As the MSC-LC intervention was specifically designed to target and reduce stigma through self-compassion practices [14], exploring changes in lung cancer stigma directly assesses the intervention’s hypothesized impact. As such, lung cancer stigma will be measured using the Lung Cancer Stigma Inventory (LCSI), which is a self-report questionnaire of lung cancer stigma that includes 25 items rated on a 5-point Likert scale from 1 (“not at all”) to 5 (“extremely”). Scores range from 25 to 125, with higher scores indicating higher lung cancer stigma [30]. The LCSI is comprised of three subscales (internalized stigma, constrained disclosure, perceived stigma), which can be summed to generate a total score [30]. Prior psychometric validation suggests that total scores of 38 or higher reflect clinically meaningful stigma levels likely associated with psychological symptom burden [1]. The LCSI was selected because of its rigorous multi-phase psychometric development process, which incorporated patient perspectives into the wording of items [30].

Depressive symptoms will be measured using the Patient Health Questionnaire-8 (PHQ-8), which is a self-report questionnaire of depressive symptoms and includes 8 items rated on a 4-point Likert scale ranging from 0 (“not at all”) o 3 (“nearly every day”) [47]. Scores range from 0 to 24, with higher scores indicating higher depressive symptom severity. Depressive symptoms are frequently reported among adults with lung cancer [48], and evaluating changes in depressive symptoms will help determine whether the intervention has broader psychological benefits, consistent with effects observed in other self-compassion trials [15].

Anxiety will be measured using the Generalized Anxiety Disorder-7 (GAD-7), which is a self-report questionnaire of anxiety symptoms and includes 7 items rated on a 4-point Likert scale ranging from 0 (“not at all”) to 3 (“nearly every day”) [49]. Scores range from 0 to 21, with higher scores indicating higher anxiety symptom severity. Given the high prevalence of anxiety in adults with lung cancer [48], assessing anxiety allows for evaluation of broader psychological improvements, aligned with prior findings from self-compassion interventions [15].

Self-compassion will be assessed using the Self-Compassion Scale, which is a self-report questionnaire of self-compassion and includes 26 items rated on a 5-point Likert scale from 1 (“never”) to 5 (“always”) [21]. Scores range from 26 to 130, with higher scores indicating higher self-compassion. The scale is comprised of six subscales (self-kindness, self-judgment, common humanity, isolation, mindfulness, and over-identification). Subscale scores are averaged (after reverse-scoring negative items), and a total mean score can be computed to reflect overall self-compassion [50]. Assessing self-compassion allows for evaluation of whether the MSC-LC practices effectively cultivate the intended emotional and cognitive skills that the intervention intends to bolster.

Spiritual well-being will be assessed using the meaning and peace subscales of the Functional Assessment of Chronic Illness Therapy - Spiritual Well-Being (FACIT-SP), which is a self-report questionnaire that includes 8 items rated on a 5-point Likert scale from 0 (“not at all”) to 4 (“very much”) [51]. Scores range from 0 to 48, with higher scores indicating higher meaning and peace during chronic illness. Assessing spiritual well-being as a secondary outcome allows for evaluation of whether the intervention fosters positive changes in a clinically important psychological outcome alongside potential reductions in distress [52].

#### Theoretically informed mediators

To inform theory-driven evaluation of intervention effects, we will include measures of hypothesized candidate mediators identified through both action theory and conceptual theory frameworks [53]. Variables targeted by action theory are those the intervention is designed to directly influence, whereas conceptual theory mediators are those theoretically associated with the outcomes [53–55]. Including these measures in the pilot trial will enable preliminary assessment of whether the intervention produces expected changes in hypothesized mechanisms and whether changes in outcomes may be explained, in part, by earlier shifts in these constructs. Although the pilot is not powered to test mediation formally, patterns of change will provide insight into whether MSC-LC functions as intended, help refine the underlying theory of change, and inform the selection of mediator measures for a future, fully powered trial.

To assess whether MSC-LC promotes broader emotional and cognitive shifts related to mindfulness and common humanity (beyond responding to difficult emotions and situations), we will measure mindfulness, rumination, social attachment, and loneliness. Mindfulness will be assessed using the 3-item observing subscale of the Five Facet Mindfulness Questionnaire (FFMQ), measuring awareness of internal and external experiences [56]. Rumination will be measured with the 12-item rumination subscale of the Rumination-Reflection Questionnaire (RRQ) to assess potential reductions in repetitive, negative self-focused thinking [57]. Social attachment will be assessed using the 4-item attachment subscale of the Social Provisions Scale to evaluate the impact of MSC-LC practices fostering connection and emotional closeness [58]. Loneliness will be measured with the 3-item UCLA Loneliness Scale-3 to determine whether the intervention reduces a sense of perceived social isolation [59].

Additionally, we will assess emotional responses to dyspnea using the breathing discomfort and emotional response subscales of the Multidimensional Dyspnea Profile (MDP) to examine whether MSC-LC’s breath-focused practices influence how participants emotionally respond to breathing difficulties [60], which are often distressing in this population [61]. To evaluate whether MSC-LC fosters more adaptive responses to breathing-related distress, we also modified the MDP to include three positively valanced emotional response options (i.e., calm, relaxed, at ease) from the PANAS-X serenity subscale [62].

To explore potential mechanisms linked to lung cancer stigma and distress, we will assess physical symptom burden and sleep disturbance. Physical symptom burden will be measured using the physical symptoms subscale of the Edmonton Symptom Assessment System–Revised (ESAS-r), which captures common cancer-related symptoms such as pain, fatigue, and nausea [63]. Sleep disturbance will be assessed using the 8-item PROMIS Sleep Disturbance short form (8b) [64], given prior evidence that sleep disruption mediates the relationship between lung cancer stigma, psychological distress, and symptom burden [5].

### Post-intervention interviews

MSC-LC participants will be invited to participate in post-intervention interviews to provide a deeper understanding of their experiences with the intervention and to capture aspects of its impact that may not be reflected in the quantitative assessments [65]. Participants in the MSC-LC condition who completed at least one intervention session will be invited to complete individual semi-structured interviews (45–60 min) conducted virtually by a trained study team member. Each interview will be audio-recorded, transcribed verbatim, and de-identified. The interview guide is designed to elicit feedback across several domains, including intervention acceptability (e.g., perceived appropriateness of intervention content, satisfaction with the intervention), reflections on the intervention and its impact, motivation and engagement factors influencing retention, group dynamics, intervention content and its tailoring to lung cancer, program structure and delivery mode, and suggestions for improvement.

### Data analysis

#### Quantitative analyses

Range checks will be conducted to identify missing, aberrant, or out-of-range values, which will be reviewed and resolved by the study team. Descriptive statistics (means, standard deviations, frequencies) will be reported for all study variables, and zero-order correlations will be examined to assess associations among outcomes and mediators. Key clinical characteristics (e.g., disease stage, smoking history) will be described across study arms and explored in relation to primary outcomes (e.g., session attendance) to inform future trial design. Analyses will be conducted using Stata v19.

The primary outcomes of interest are feasibility and acceptability indices; clinical and psychosocial outcomes will be considered secondary exploratory outcomes. Progression criteria were established a priori to classify outcomes as feasible, in need of amendment, or not feasible based on predefined thresholds. Feasibility will be evaluated using descriptive statistics. The intervention will be considered feasible to deliver if at least 60% of participants in the MSC-LC condition attend six or more sessions, reflecting meaningful engagement while accounting for common attendance barriers in oncology populations; it will be considered not feasible if 33% or fewer meet this threshold. Recruitment will be considered feasible if at least 50% of eligible participants (with lung cancer diagnosis confirmed via medical record) agree to enroll and be randomized, and not feasible if recruitment uptake is 33% or less. Retention will be considered feasible if at least 70% of participants remain in the study through the post-intervention assessment and not feasible if 50% or fewer are retained. Treatment fidelity adherence will be considered adequate if ≥80% of predefined intervention components are delivered per session, ensuring that the intervention is delivered with sufficient integrity, and not considered feasible if fidelity adherence is 50% or less. Fidelity via interventionist competence will be deemed sufficient if the mean MSC TAC rating across the three sampled sessions per cohort and per interventionist is greater than or equal to 4 (“competent”) with no individual domain less than 3 (“advanced beginner”). We will also report the proportion of ratings at or above 5 (“proficient”) as an indicator of intervention quality.

As this is a pilot trial designed to assess feasibility and explore preliminary signals of intervention effects, analyses of patient-reported outcomes will be descriptive and exploratory, based on available data. For each patient-reported outcome, we will report descriptive statistics and 95% confidence intervals, where relevant, at each assessment timepoint (baseline, mid-intervention, post-intervention, follow-up) separately by group. For interpretability, we will also report the proportion of participants at each timepoint who meet or exceed suggested clinical cutoffs for select measures (e.g., LCSI ≥ 38, PHQ-8 ≥ 10, GAD-7 ≥ 10), as well as the proportion demonstrating clinically meaningful change (e.g., moving from above to below threshold). These findings will be used to refine theory-driven hypotheses and inform analytic strategies for a future trial. Exploratory analyses of potential mechanisms (e.g., changes in mindfulness, loneliness) will not be formally modeled in this pilot trial; however, descriptive patterns in these hypothesized mediators will be reported.

No interim analyses or formal stopping rules are planned given the exploratory and pilot nature of the trial. The PI will make the final decision regarding early study termination in consultation with the IRB if safety concerns or unanticipated issues arise. Given the small sample size, statistical models will be applied in an exploratory manner to examine patterns of change. Exploratory effect size estimates will be reported to inform variance estimation and plausible effect size ranges for planning a future definitive trial, rather than to evaluate efficacy [66]. Estimates will be interpreted cautiously, recognizing that the stability of effect sizes is limited in this pilot study context. Missing data will be summarized descriptively (extent and patterns across assessment timepoints) to contextualize and interpret feasibility outcomes. Findings will be used to refine hypotheses and analytic strategies for subsequent, adequately powered trials.

#### Qualitative analyses

Qualitative data from post-intervention interviews will be analyzed using thematic content analysis. Two trained research team members will independently read the transcripts and inductively generate initial codes. The research team will meet to discuss code content, resolve discrepancies, and collaboratively develop a codebook. Once finalized, transcripts will be coded using NVivo software. Coders will meet regularly to ensure consensus and refine emergent themes. The analytic focus will include participants’ acceptability of and satisfaction with the MSC-LC intervention, perceived impact on stigma and self-compassion, and feedback on program structure, content, and delivery. Responses from participants in the highest tertile of stigma reduction (i.e., greatest improvement) and the lowest tertile of change in stigma scores (i.e., least improvement) will be analyzed to explore variability in intervention impact and to identify potential barriers and facilitators to engagement. Rigor, transparency, and trustworthiness will be supported through maintenance of an audit trail, documented codebook development and refinement, reflexive coding practices, and consistent application of the final codebook across and within transcripts.

#### Mixed-methods integration

Quantitative and qualitative data will be integrated at the interpretation phase using a convergent design. A joint display table will be developed to visually compare quantitative findings (e.g., change scores, clinical thresholds, correlations) with qualitative themes for key outcomes (e.g., stigma reduction, psychological distress, self-compassion). This matrix will allow for cross-validation of findings, exploration of convergence/divergence, and triangulation of insights. For example, it may be informative to integrate data showing a divergence between stigma score changes and perceived stigma narrative shifts. Qualitative data will also be used to help explain unexpected patterns in the quantitative findings and to identify intervention impacts not captured by standard measures. This mixed-methods integration will inform interpretation of pilot findings, optimization of MSC-LC content, and planning for future randomized trials.

## Discussion

Given the pervasive and robust associations between stigma, psychological distress, quality of life, and physical symptoms in this population [1–3, 5, 7, 22], the development and evaluation of patient-centered interventions specifically aimed at stigma reduction are urgently needed. This pilot randomized controlled trial represents an important step in addressing the unmet psychosocial needs of people with lung cancer by targeting the reduction of lung cancer stigma through a novel, tailored MSC-LC intervention [11, 12, 14]. MSC-LC builds upon a well-established evidence base for MSC interventions by adapting content, delivery format, and practices to the unique challenges and symptom burden experienced by people with lung cancer, such as dyspnea and fatigue, while explicitly addressing stigma and its emotional sequelae.

The use of a mixed-methods design, incorporating both quantitative outcome assessments and in-depth qualitative interviews, allows for a nuanced evaluation of MSC-LC’s feasibility, acceptability, and preliminary efficacy. Quantitative measures will provide important data on changes in lung cancer stigma, self-compassion, depression, anxiety, and spirituality, offering early indicators of potential clinical benefit. Additionally, qualitative interviews will enrich understanding of the intervention’s meaningful components, participant engagement, and mechanisms of change that may not be captured by quantitative assessments alone.

This study addresses a significant gap in psychosocial oncology, as no prior randomized controlled trials have evaluated a patient-focused intervention designed to reduce lung cancer stigma. The virtual, group-based format is designed to enhance accessibility for a geographically diverse population experiencing high symptom burden and complex medical needs, reducing common barriers to psychosocial care such as transportation challenges, bothersome physical symptoms, and isolation. In particular, virtual delivery may help reduce disparities in access to psychosocial interventions by reaching patients in rural areas and those with mobility limitations who might otherwise be excluded from in-person care. By fostering self-kindness, mindfulness, and connection within a supportive group context, MSC-LC aims to empower participants to better cope with stigma-related distress and to enhance overall emotional well-being. Beyond stigma reduction, cultivating self-compassion may also serve as a foundation for improved self-regulation in the cancer care context with potential to support treatment adherence, patient–provider communication, and engagement in care.

The investigators plan to disseminate study findings through reporting of results in peer-reviewed publications, presentations at scientific and community meetings, and a summary that is shared back with participants who indicated interest in learning about study findings. Access to study data, materials, and analytic code will be provided by reasonable request made to the Principal Investigator, as allowable by regulations for the protection of human subjects and data management.

### Limitations and strengths

This study has several limitations. The study includes a geographically diverse sample; however, there may be bias toward participants who are comfortable with group-based interventions and may already be open to participating in mindfulness-based approaches. Reliance on self-reported measures and the absence of an active comparator may affect interpretation of outcomes. While virtual delivery improves accessibility, it also poses digital challenges, limiting participation to those with reliable broadband and access to necessary technology such as a camera, microphone, and computer or smartphone. Recruitment strategies that rely on online platforms may result in greater participation among individuals with higher levels of digital access and comfort. As such, recruitment sources will be tracked and reported to characterize the sample and inform strategies to enhance access and broadly representative inclusion in future trials. MSC-LC primarily addresses internalized stigma and constrained disclosure, rather than systemic or provider-level factors, highlighting the need for complementary, multilevel stigma reduction strategies to achieve broader impact. The small sample size restricts the applicability of the proposed analyses and limits the confidence with which results can be generalized. Effect estimates should therefore be interpreted as preliminary signals rather than definitive outcomes. This limitation underscores the pilot nature of the study, which is intended to guide the design of a larger trial. Finally, the qualitative sample may not fully capture all participant perspectives, particularly those who disengage midway through the trial. To address this, we will attempt to contact participants who discontinued their participation to invite them for interviews about their reasons for withdrawal and any barriers they experienced.

This study’s strengths include a well-justified and theoretically-driven intervention with a rigorous methodological plan that specifies a priori standards for determining feasibility and acceptability. Additionally, the study’s geographically diverse recruitment enhances the potential for broader generalizability beyond a single location and facilitating future scaling. Finally, the mixed-methods design allows for a comprehensive evaluation of both quantitative outcomes and in-depth participant experiences.

## Conclusions

If demonstrated to be feasible and acceptable, MSC-LC has the potential to fill an important gap in lung cancer care by providing a scalable, patient-centered intervention to reduce stigma and enhance self-compassion. Findings from this pilot trial will inform refinements to the intervention protocol, support the design of a fully powered efficacy trial, contribute to the growing evidence base for use of mindfulness and compassion-based interventions in oncology populations, and lay the groundwork for implementation science questions related to navigator-facilitated delivery, integration within routine oncology workflows, and scalability across diverse health system settings. Future research may explore evaluating the impact of MSC-LC on healthcare engagement outcomes, extending MSC-LC to caregivers and family members, and integrating MSC-LC with provider-level stigma reduction strategies to address stigma comprehensively. Ultimately, MSC-LC holds strong promise as an innovative intervention that empowers people with lung cancer to navigate stigma, build resilience, and enhance their emotional well-being and quality of life amid complex challenges. This pilot trial aims to lay the groundwork for next steps toward efficacy testing and broader dissemination and implementation efforts, catalyzing a transformative shift in how stigma is addressed in lung cancer care and advancing meaningful improvements in patients’ well-being and quality of life.

## Data Availability

De-identified study data and materials are available upon reasonable request from the corresponding author.

## Abbreviations

MSC-LC: Mindful Self-Compassion for Lung Cancer
MSC: Mindful Self-Compassion
RCT: Randomized Controlled Trial
CTL: Control (enhanced standard-of-care waitlist control group)
SPIRIT: Standard Protocol Items Recommendations for Interventional Trials
IRB: Institutional Review Board
LCSI: Lung Cancer Stigma Inventory
NCI: National Cancer Institute
NCCN: National Comprehensive Cancer Network
C-TUQ: Cancer Patient Tobacco Use Questionnaire
PROMIS: Patient-Reported Outcomes Measurement Information System
PHQ-8: Patient Health Questionnaire-8
GAD-7: Generalized Anxiety Disorder-7
FACIT-SP: Functional Assessment of Chronic Illness Therapy - Spiritual Well-Being
FFMQ: Five Facet Mindfulness Questionnaire
RRQ: Rumination-Reflection Questionnaire
MDP: Multidimensional Dyspnea Profile
PANAS-X: Positive and Negative Affect Schedule
ESAS-r: Edmonton Symptom Assessment System–Revised
REML: Restricted Maximum Likelihood Estimation
